# Housekeeping gene dysregulation in psoriasis: integrative multi‐cohort and single‐cell analysis reveals keratinocyte‐centric molecular mechanisms and diagnostic biomarkers

**DOI:** 10.3389/fimmu.2025.1601705

**Published:** 2025-09-01

**Authors:** Hao Tang, Jiacheng Wang, Shuhao Zhang, Guanglong Feng, Xiangshu Cheng, Xin Meng, Rui Chen, Jiaqi Wang, Yongshuai Jiang, Ruijie Zhang, Wenhua Lv

**Affiliations:** ^1^ College of Bioinformatics Science and Technology, Harbin Medical University, Harbin, China; ^2^ Department of CT Diagnosis, The Second Affiliated Hospital of Harbin Medical University, Harbin, China

**Keywords:** housekeeping genes, keratinocyte, machine learning models, psoriasis, single-cell transcriptomics

## Abstract

**Background:**

Psoriasis is a chronic immune-mediated skin disease driven by the interleukin-23/interleukin-17 cytokine axis, yet its immunopathogenesis remains incompletely understood. Housekeeping genes, traditionally considered stably expressed across tissues and cell types, have not been systematically investigated for their role in psoriasis. Here, we aimed to identify psoriasis-associated housekeeping genes and explore their molecular mechanisms and clinical implications.

**Methods:**

We integrated multi-cohort data and identified psoriasis-associated housekeeping genes using weighted gene co-expression network analysis combined with differential expression analysis. Single-cell transcriptomic analysis was performed to identify cell-type specific expression patterns, while ligand-receptor interaction analysis was applied to evaluate pathway activation and interactions with downstream target genes. In addition, multiple diagnostic models were established for psoriasis detection.

**Results:**

We identified 34 housekeeping genes associated with psoriasis and observed that the co-expression relationships between six genes (APOL2, DCUN1D3, UBE2F, HIGD1A, PPIF, and STAT3) and known psoriasis-related genes differed significantly between diseased and healthy individuals. Furthermore, single-cell transcriptomic analysis revealed that these housekeeping genes were differentially expressed primarily in basal, spinous, supraspinous, and proliferating keratinocytes. Ligand-receptor interaction analysis demonstrated significant activation of the IL - 17, IL - 6, and midkine (MK) pathways within keratinocyte subpopulations, which led to the upregulation of STAT3, EIF5A, and RAN, thereby promoting keratinocyte hyperproliferation and enhancing immune reactivity. Finally, among the various diagnostic models developed, the averaged neural network (avNNet) model emerged as the best performer, achieving over 90% classification accuracy across multiple independent datasets. Moreover, its scores were strongly correlated with the Psoriasis Area and Severity Index (correlation coefficient = 0.74, P = 4.4e-47).

**Conclusions:**

This study redefines housekeeping genes as dual-function regulators in psoriasis pathogenesis, with the avNNet model enabling clinical translation of these molecular insights toward precision-targeted therapies and biomarker-based management strategies.

## 1 Introduction

Psoriasis is a common chronic, non-contagious skin disease with a globally variable prevalence, ranging from 0.09% to 11.43% across different countries. It is estimated to affect at least 100 million people worldwide, making it a significant global health concern (1). The etiology of psoriasis is complex, involving both genetic and environmental factors. Triggers such as infections, sunburn, and smoking can contribute to disease onset and exacerbation (2), while genetic predisposition remains a major risk factor. Genome-wide association studies (GWAS) have identified over 80 genetic risk loci associated with psoriasis, yet these variants collectively account for only approximately 30% of the disease’s heritability (3). Clinical studies have demonstrated that hyperactivation of the IL - 23/IL-17 axis is a key driver of psoriasis pathogenesis. Additionally, IL - 36, a cytokine produced by keratinocytes, has been strongly implicated in disease progression (4). The hallmark characteristics of psoriasis include epidermal hyperplasia and immune cell infiltration into the dermis, with keratinocyte hyperproliferation being the primary cause of epidermal thickening. Keratinocytes play a crucial role in both the initiation and maintenance of psoriasis. In response to external triggers, they release nucleotides and antimicrobial peptides, which activate dendritic cells involved in the early stages of disease development. Once activated by various pro-inflammatory signals, keratinocytes undergo excessive proliferation and secrete chemokines that recruit leukocytes and inflammatory mediators, thereby amplifying the inflammatory response. As key components of the innate immune system, keratinocytes not only sustain the inflammatory milieu but also actively contribute to disease progression (5).

Housekeeping genes (HKGs) are universally expressed in cells and play a fundamental role in maintaining essential physiological functions. Under normal conditions, they are constitutively expressed across all cell types, regardless of their specific function, developmental stage, or cell cycle phase (6). Due to these characteristics, HKGs are widely used as internal controls in molecular biology and computational experiments. However, recent studies have revealed a strong association between HKGs and complex diseases. For instance, SURF4 has been shown to inhibit myeloid differentiation and suppress cell death in myeloid leukemia cells by negatively regulating the STING-TBK1-STAT6 axis (7). Similarly, STAT3 activation has been implicated in the progression of breast cancer (8), while RAB5A expression is upregulated in an m6A-YTHDF2-dependent manner by ALKBH5, promoting colorectal cancer development (9). In the context of psoriasis, disease-associated variants in the ERAP1 gene have been found to increase susceptibility in individuals carrying the HLA-C risk allele (10). However, despite emerging evidence linking HKGs to disease mechanisms, no studies have systematically investigated the impact of their aberrant expression on the progression of psoriasis.

Psoriasis treatment has long been an important topic, as conventional therapies often fail to prevent relapse. In recent years, the development of biologic agents has made substantial progress, enabling long-term remission of lesions (11). Objective diagnosis and evaluation are critical when selecting treatment strategies and assessing therapeutic outcomes. Currently, psoriasis diagnosis and classification are primarily based on clinical features and patient history, supplemented when necessary by dermoscopy and histopathological analysis (12). However, the diagnostic process can be influenced by clinician experience and remains somewhat subjective. Therefore, as treatment modalities continue to advance, improving the accuracy and convenience of diagnosis and evaluation becomes increasingly important. Molecular diagnostic techniques, a key component of precision medicine, have achieved significant progress in recent years and are now applied clinically for diagnosing genetic diseases and cancers—particularly by detecting changes in gene expression at the DNA and RNA levels to guide optimal treatment selection (13). These techniques rely on advanced technologies such as microarrays and next-generation sequencing. They have also been widely applied in psoriasis research; for example, GWAS in Chinese populations have identified several genes associated with psoriasis susceptibility (14). Accurate diagnosis and assessment of psoriasis can facilitate more appropriate treatment decisions, although the clinical application of these molecular technologies is still evolving. With the widespread adoption of microarray and RNA sequencing technologies, vast amounts of psoriasis-related data have been accumulated in public repositories like the Gene Expression Omnibus (GEO). Therefore, developing an accurate evaluation platform using these existing RNA expression datasets is of great importance.

In this study, we integrated bulk and single-cell transcriptomic data to investigate the impact of aberrant housekeeping gene expression on psoriasis progression. Candidate driver HKGs associated with psoriasis were identified through differential expression analysis combined with weighted gene co-expression network analysis (WGCNA). Biological pathways influenced by these genes were explored using enrichment analysis. Co-expression analysis, combined with immune infiltration assessment, was performed to determine the predominant cell types affected by HKGs. Additionally, ligand-receptor interaction analysis was conducted to explore cell-cell communication and elucidate how these interactions influence housekeeping gene expression in keratinocytes, thereby contributing to disease progression. Finally, we applied machine learning approaches to construct robust predictive models for psoriasis assessment, aiming to improve diagnostic accuracy and disease monitoring.

## 2 Materials and methods

### 2.1 Collection and processing of bulk RNA datasets

All datasets used in this study were retrieved from the GEO database. Initially, datasets were screened based on sample size, sample type, and sequencing platform. To ensure the reliability of the analysis and to minimize the impact of confounding factors such as drug treatment and tissue type on gene expression, only pre-treatment skin tissue samples were included, with the exception of GSE117468. A summary of the datasets is provided in **Table 1**. Batch effects were corrected using the “removeBatchEffect” function from the limma R package (15), and principal component analysis (PCA) was performed using the factoextra R package to assess the preprocessed data. Differential expression analysis was conducted with the limma R package, considering genes with an adjusted p-value of less than 0.05 and an absolute log fold change (logFC) greater than 0.5 as differentially expressed.

**Table 1:** Brief information of GEO datasets.

GEO accession	Platform	Tissue	Size	Treatment	Source type
GSE54456	GPL9052	Human skin	174	No treatment	RNA-seq
GSE14905	GPL570	Human skin	54	No treatment	Array
GSE226244	GPL570	Human skin	41	No treatment	Array
GSE182740	GPL570	Human skin	15	No treatment	Array
GSE121212	GPL16791	Human skin	66	No treatment	RNA-seq
GSE109248	GPL10558	Human skin	31	No treatment	Array
GSE66511	GPL16288	Human skin	24	No treatment	RNA-seq
GSE80047	GPL13158	Human skin	50	No treatment	Array
GSE53431	GPL10558	Human skin	24	No treatment	Array
GSE117468	GPL570	Human skin	565	Brodalumab Ustekinumab	Array
GSE173706	GPL24676	Human skin	22	No treatment	scRNA-seq
GSE192867	GPL23126	Human PBMC	72	No treatment	Array

### 2.2 Collection and processing of single-cell datasets

In this study, single-cell RNA sequencing data from psoriasis patients and controls were obtained from the GEO database (accession number GSE173706), uploaded by Ma Feiyang et al (16). The dataset comprises 14 psoriasis samples and 8 control samples. A summary of the datasets is provided in **Table 1**. Analysis was performed using the Seurat R package (version 5.1.0) (17). Low-quality cells were filtered out by removing cells that expressed fewer than 100 genes, more than 5,000 genes, fewer than 500 transcripts, or in which mitochondrial genes accounted for more than 10% of total transcripts. Normalization and variable feature identification were performed independently for each sample. Batch effects were subsequently corrected and the samples integrated using the “RunHarmony” function from the Harmony R package (18). After clustering, dimensionality reduction was achieved via Uniform Manifold Approximation and Projection (UMAP). Cluster-specific marker genes were identified using the “FindAllMarkers” function, and cell types were manually annotated based on the original publication and additional literature, with cells of unidentified types excluded from further analysis. Differential expression analysis between normal skin (NS) and lesional psorisais (PP) was conducted using the “run_de” function from the Libra R package.

### 2.3 Human housekeeping gene list

In this study, HKGs were defined based on the comprehensive re‐analysis by Eisenberg and Levanon (6). In their work, HKGs were identified as genes that are ubiquitously expressed across a wide range of human tissues and cell types, reflecting their essential roles in maintaining core cellular functions. Specifically, Eisenberg and Levanon surveyed publicly available high‐throughput sequencing datasets encompassing diverse normal human tissues to pinpoint genes with consistently stable expression profiles. The selection criteria for housekeeping genes (HKGs) mandated: (1) ubiquitous detection across all examined tissues, (2)low inter-tissue variability (standard deviation of log_2_(RPKM) < 1), and (3) no extreme outlier expression (no tissue with expression differing by ≥4-fold from the average). Genes were designated as HKGs when ≥50% of exons in any RefSeq transcript satisfied these parameters, yielding the 3,804 high-confidence HKG set that served as our analytical framework.

### 2.4 Weighted gene co-expression network analysis

To elucidate the relationship between gene expression patterns and disease states, we conducted Weighted Gene Co-expression Network Analysis on the differentially expressed genes using the WGCNA R package (19). First, we calculated a gene similarity matrix based on Pearson correlation coefficients. Next, we constructed an adjacency matrix by selecting an appropriate soft-thresholding parameter (β) to accentuate the connections between highly correlated gene pairs. The resulting adjacency matrix was then transformed into a Topological Overlap Matrix (TOM), which quantifies the network connectivity of genes and provides a measure of dissimilarity (1-TOM). Hierarchical clustering was subsequently performed on the TOM to group genes into modules, with module detection carried out using dynamic tree cutting methods. Finally, correlations between modules and disease states were calculated to identify those modules that were significantly associated with the investigated diseases.

### 2.5 Functional enrichment analysis of genes

To elucidate the biological functions of the gene set of interest, we performed functional annotation using the clusterProfiler R package (20). Both Gene Ontology (GO) and Kyoto Encyclopedia of Genes and Genomes (KEGG) enrichment analyses were conducted. An adjusted p-value of <0.05 was used as the threshold for statistical significance.

### 2.6 Immune infiltration analysis

Single‐cell data from GSE173706 was used as the reference matrix, while RNA-seq data from GSE54456 served as the mixture file. First, CIBERSORTx (https://cibersortx.stanford.edu/) was employed to generate a signature matrix from the single‐cell RNA sequencing data (21). Subsequently, the tool was used to estimate the abundance of nine distinct cell types.

### 2.7 Inference of intercellular interactions

CellChat (v2.1.2) was employed to analyze receptor-ligand interactions (22). Except for keratinocytes, which were further divided into subclusters, all other cells were annotated according to their primary cell types. To compare cell communication between the PP and NS groups, each sample group was analyzed separately, and the number of interactions between different cell types was quantified. Furthermore, to investigate how other cells influence keratinocytes by modulating the expression of key HKGs, these HKGs were designated as target genes. Subsequently, the nichenetr package (v2.2.0) was used to identify the connections between ligands and their target genes (23). Finally, we used Cytoscape (v3.7.2) to construct and visualize the ligand-to-target gene regulatory network (24).

### 2.8 Construction of machine learning models

Using the caret R package (25), we constructed machine learning models with the 34 identified key HKGs serving as features. The dataset was randomly partitioned, with 80% of the samples assigned to the training set and the remaining 20% to the testing set. In total, five distinct models were developed: Random Forest (rf), Naïve Bayes, Averaged Neural Network (avNNet), Support Vector Machine with Radial Basis Function kernel (svmRadial), and Generalized Linear Model Network (glmnet).

To assess the classification performance of these models, we computed several metrics, including recall, precision, accuracy, F1 score, and the area under the receiver operating characteristic curve (AUC). The AUC was calculated using the pROC R package, while the other metrics were generated via the “confusionMatrix” function from the caret R package.

Code Availability: The R scripts used for model training, performance evaluation, and figure generation are provided in **Supplementary Data S1**. This archive includes a README file that details all software dependencies and provides step-by-step instructions to reproduce our results.

## 3 Results

### 3.1 34 key housekeeping genes associated with psoriasis identified through WGCNA and differential analysis

**Figure 1:**
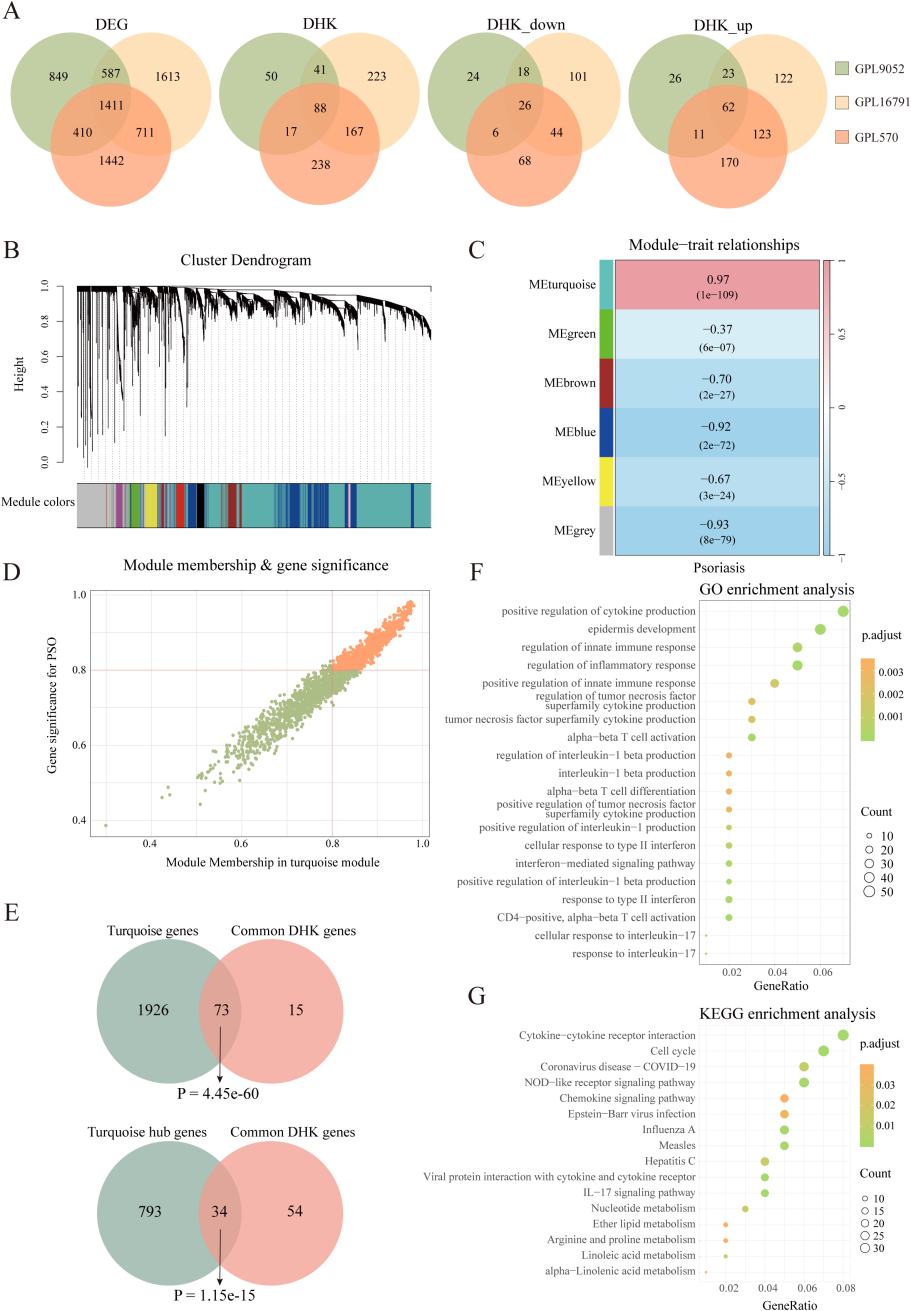
Identification of psoriasis related housekeeping genes. **(A)** Venn diagram illustrating the overlap of differentially expressed genes between various datasets, including the number of differentially expressed housekeeping genes. **(B)** Cluster dendrogram and module assignment for the GPL9052 cohort. **(C)** Heatmap showing the association between gene expression modules and phenotypic traits. **(D)** Scatter plot showing correlation of genes within modules to modules and to psoriasis traits. **(E)** Venn diagram shows the overlap of 88 DHKs with the turquoise module genes and the turquoise module hub genes. **(F)** GO enrichment analysis. **(G)** KEGG enrichment analysis. DEG, Differentially expressed gene; DHK, Differentially expressed housekeeping gene; GO, Gene Ontology; KEGG, Kyoto Encyclopedia of Genes and Genomes.

To systematically identify key HKGs linked to psoriasis pathogenesis, we integrated transcriptomic datasets from GSE14905, GSE226244, and GSE182740. PCA confirmed effective batch effect correction after dataset merging (**Supplementary Figures S1A, B**). The integrated dataset was annotated according to its sequencing platform. Differential expression analysis was conducted separately on three datasets: GPL570 (integrated dataset), GPL9052 (GSE54456), and GPL16791 (GSE121212). We identified 3,257, 3,974 and 4,322 differentially expressed genes (DEGs) between psoriasis patients and healthy controls for GPL9052 dataset, GPL570 dataset and GPL16791 dataset respectively. The intersection across these three platforms resulted in 1,411 common DEGs. Subsequently, we intersected the common DEGs with the human housekeeping gene list. We identified 196, 570 and 519 differentially expressed HKGs in the GPL9052, GPL570 and GPL16791 datasets, respectively Among these, 88 HKGs were consistently differentially expressed across all three platforms, with 62 upregulated and 26 downregulated (**Figure 1A**).

Given that the GPL9052 dataset had the largest sample size (92 psoriasis patients and 82 healthy controls), we selected it for subsequent analyses. We used its 3,257 DEGs to construct a weighted gene co-expression network. Based on the scale-free topology fitting index and average connectivity, a soft-thresholding power of 16 was chosen (**Supplementary Figures S1C, D**). Dynamic tree cutting and average hierarchical clustering identified six modules (**Figure 1B**). Module–trait correlation analysis revealed that the turquoise module was most strongly associated with psoriasis (correlation coefficient = 0.97, p = 1e-109) (**Figure 1C**). Nearly 83% (73/88) of HKGs appear in the turquoise module, underscoring a close relationship between these genes and psoriasis progression (**Figure 1E**).

We then calculated the module membership (MM) for the turquoise module and the gene significance (GS) for disease traits, identifying 827 genes significantly associated with psoriasis that exhibited high MM (>0.8) and high GS (>0.8), including 34 HKGs (**Figures 1D, E**). KEGG and GO enrichment analyses were subsequently performed on these 827 genes, focusing on pathways that involved HKGs. GO analysis indicated that these HKGs were involved in processes closely related to psoriasis, including inflammatory response, epidermis development, and IL - 17 signaling. In the KEGG analysis, the HKGs were primarily enriched in cell cycle and chemokine signaling pathways (**Figures 1F, G**).

In summary, through combined differential expression and WGCNA analyses, we identified 34 HKGs that are closely associated with psoriasis progression. The enrichment results further indicate that these HKGs play a widespread role in the disease’s pathogenesis.

### 3.2 Significant alterations in the co-expression relationships between key housekeeping genes and psoriasis-associated genes

In the GPL9052 dataset, we calculated the correlations between 34 HKGs and all other genes separately in the disease and control groups. The correlation coefficients were categorized into eight intervals: strongest negative correlation (−1 to −0.75), strong negative correlation (−0.75 to −0.5), weak negative correlation (−0.5 to −0.25), weakest negative correlation (−0.25 to 0),weakest positive correlation (0 to 0.25), weak positive correlation (0.25 to 0.5), strong positive correlation (0.5 to 0.75), and strongest positive correlation (0.75 to 1) (26). We then examined how these co-expression relationships changed from controls to psoriasis cases (**Table 2**). Notably, the correlation coefficients for 13 gene pairs shifted from strong negative to weak positive, 51 gene pairs transitioned from weak negative to strong positive, one gene pair changed from weak negative to strongest positive, and 15 gene pairs shifted from weakest negative to strongest positive. These shifts spanned at least four intervals from controls to cases. Additionally, 59 gene pairs that changed from weakest positive to strongest positive were also included in our analysis. We infer that these 139 gene pairs alterations in co-expression relationships are induced by psoriasis.

**Table 2:** The number of gene pairs in eight sections for psoriasis cases and controls.

Pso / HC	Pso [-1,-0.75]	Pso (-0.75,-0.5]	Pso (-0.5,-0.25]	Pso (-0.25,0]	Pso (0,0.25]	Pso (0.25,0.5]	Pso (0.5,0.75]	Pso (0.75,1]
HC [-1,-0.75]	41	339	49	2	0	0	0	0
HC (-0.75,-0.5]	179	7912	8096	1531	198	13	0	0
HC (-0.5,-0.25]	95	8497	28130	22011	7256	991	51	1
HC (-0.25,0]	4	3070	34302	86217	66512	13967	1055	15
HC (0,0.25]	0	595	12630	63063	80636	32892	4540	59
HC (0.25,0.5]	0	15	1282	8436	20658	23494	9995	382
HC (0.5,0.75]	0	0	85	328	1555	7127	9263	791
HC (0.75,1]	0	0	1	6	8	109	893	269

Among these 139 gene pairs with substantial changes, several interesting pairs were identified. For example, APOL2 paired with SERPINB13; DCUN1D3 paired with DEFB103A, DEFB103B, IL1F5 (IL36RN), IL1F6 (IL36A), and IL1F9 (IL36G); HIGD1A paired with SERPINB3; PPIF paired with DEFB103A, DEFB103B, and IL1F6; STAT3 paired with IL1F5, IL1F9, SERPINB7, and S100A7A; and UBE2F paired with DEFB103A, DEFB103B, IL1F9, SERPINB1, SERPINB3, SERPINB4, and S100A12 (**Supplementary Figure S2A**). Notably, SERPINB1, SERPINB3, SERPINB4, SERPINB7, and SERPINB13 belong to the serine protease inhibitor family, which play critical roles in maintaining skin barrier function, immune regulation, and inflammatory responses (27). DEFB103A and DEFB103B are members of the human β-defensin gene family and encode small antimicrobial peptides that are pivotal in innate immunity, countering a range of pathogens including bacteria, fungi, and certain viruses (28). S100A7A and S100A12, part of the S100 protein family, are primarily involved in inflammatory processes and are upregulated in psoriasis, thereby exacerbating inflammation (29). IL1F5, IL1F6, and IL1F9 belong to the interleukin-1 family and are centrally involved in mediating inflammation and immune responses (30). Although IL1F5 acts as an antagonist of the IL - 36 receptor, previous studies have consistently observed its high expression in psoriasis (31). Among the 34 key HKGs identified, the co-expression relationships with previously reported psoriasis-associated pathogenic genes shifted from negative to positive (or from weakest to strongest correlations). Given that these pathogenic genes are aberrantly expressed in keratinocytes (5, 16, 32), our findings suggest that these HKGs may influence the progression of psoriasis by modulating the biological functions of keratinocytes.

### 3.3 Differential expression of key housekeeping genes primarily in keratinocytes

**Figure 2:**
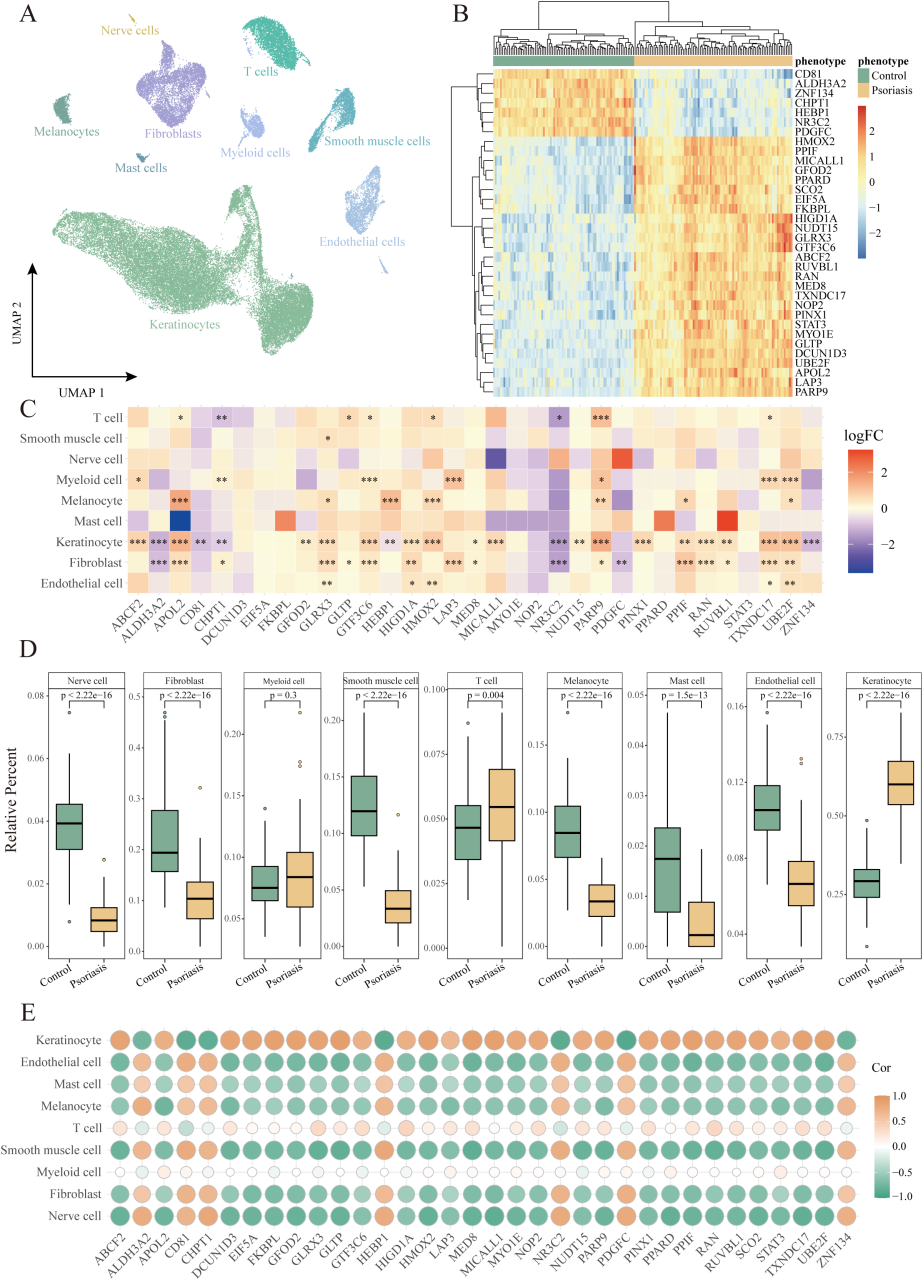
Identification of cell populations with differential expression of housekeeping genes in psoriasis. **(A)** UMAP plot showing 48,287 cells colored by cell types. **(B)** Heatmap of the expression of 34 housekeeping genes in the GPL9052 cohort. **(C)** Heatmap of differential expression of housekeeping genes in different cell types. *P<0.05, **P<0.01, ***P<0.001. **(D)** Box plot demonstrating comparison of relative abundance of different cell types between GPL9052 cohort psoriasis and controls. **(E)** Dot plot showing the correlation between relative cell abundance and gene expression.

To validate whether the identified key HKGs are differentially expressed in keratinocytes, we analyzed single-cell transcriptomic data derived from skin tissues of psoriasis patients. This dataset comprised 14 psoriasis patients and 8 healthy controls. After quality control, a total of 48,287 high-quality cells were retained. Using the Seurat package, we identified nine distinct cell types, including keratinocytes, fibroblasts, T cells, endothelial cells, smooth muscle cells, neurons, myeloid cells, mast cells, and melanocytes (**Figure 2A**; **Supplementary Figure S3A**) (16, 33).

Differential expression analysis comparing lesional skin to healthy controls revealed that, within keratinocytes, 8 key HKGs were downregulated and 25 genes were upregulated, with one gene undetected. Notably, the expression patterns of these key HKGs in keratinocytes from the single-cell data were highly consistent with those observed in bulk transcriptomic analyses. The majority of key HKGs (32 out of 34) showed consistent expression in keratinocytes between bulk and single-cell transcriptomic data—7 genes were downregulated and 25 genes were upregulated (**Figures 2B, C**), with only NOP2 displaying inconsistent expression and SCO2 undetected in the single-cell dataset. Although a few genes did not reach statistical significance in the single-cell analysis, their expression trends remained consistent with the bulk data.

Furthermore, cell type deconvolution analysis using CIBERSORTx indicated that keratinocytes constituted the largest proportion among all cell types, with a significantly higher proportion observed in psoriasis patients compared to healthy controls (**Figure 2D**). This finding is in line with the clinical characteristics of psoriasis, which is marked by hyperproliferation of keratinocytes and consequent epidermal thickening. Additionally, correlation analysis between the key HKGs and various cell types revealed that the upregulated genes were significantly positively correlated with keratinocytes, whereas the downregulated genes exhibited a significant negative correlation (**Figure 2E**).

Collectively, these results indicate that the differential expression of the identified key HKGs is primarily driven by changes in keratinocytes.

### 3.4 IL-17A regulates the expression of four HKGs in keratinocyte subpopulations

**Figure 3:**
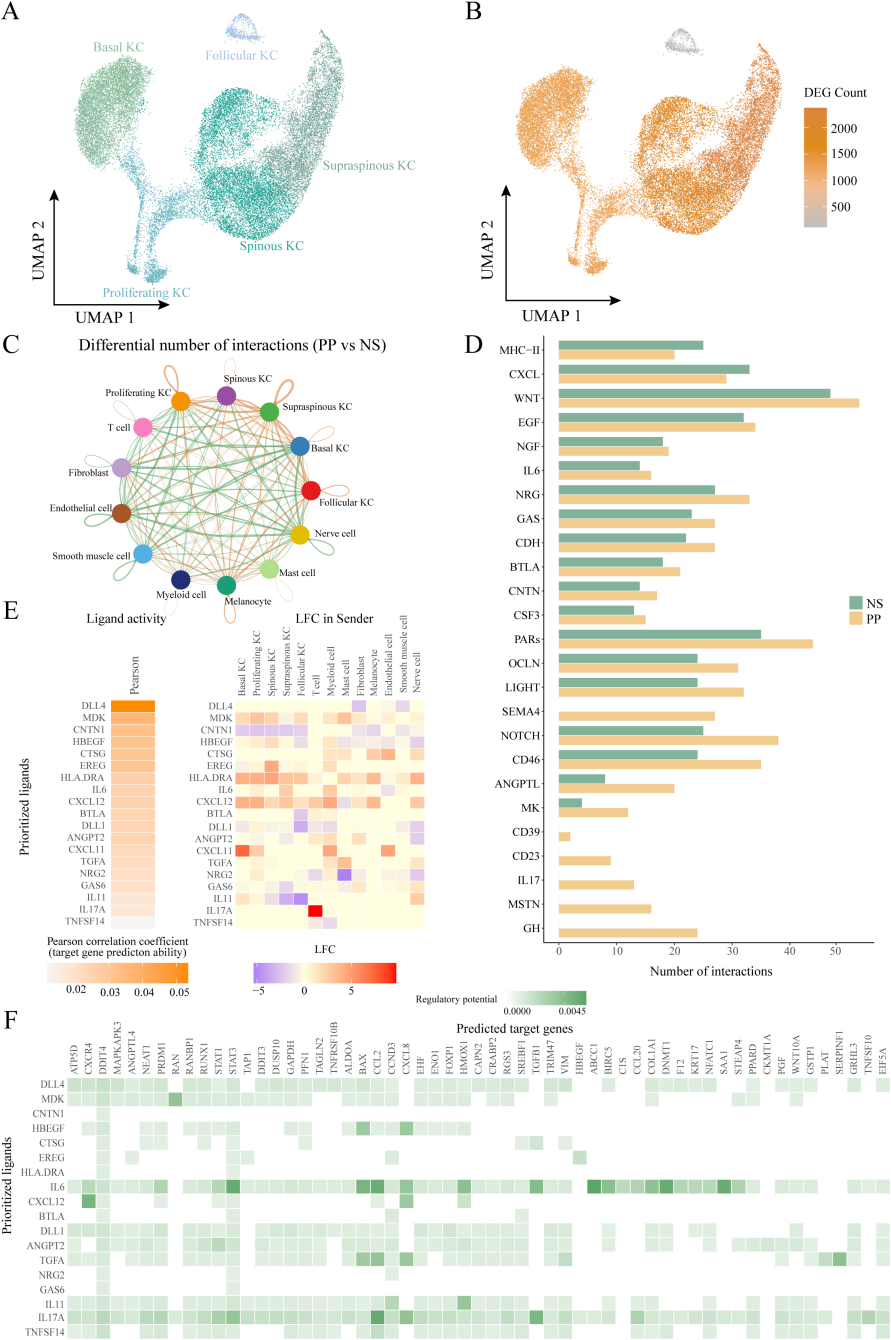
Ligand-receptor interactions of other cells with keratinocyte subtypes. **(A)** UMAP plot showing 29489 keratinocytes colored by cell types. **(B)** UMAP plot showing the number of differentially expressed genes in keratinocyte subtypes in diseased and healthy skin. **(C)** Circle plot comparing the number of ligand-receptor interactions between cell types of lesion and control skin. **(D)** Bar graph depicting pathways with significantly more cellular communication in diseased skin than in control. **(E)** NicheNet ligand-receptor analysis: heatmap of ligand activity (left) and differential expression (right) of ligands in different cell types. **(F)** NicheNet ligand-receptor analysis: matrix showing potential targets of ligands and their regulatory potential in four keratinocyte subtypes. KC, keratinocyte; PP, lesional psoriasis; NS normal skin.

To further elucidate the role of HKGs in keratinocytes, we adopted the keratinocyte subcluster classification defined by Francis et al (33). Keratinocytes were categorized into five subpopulations: follicular (KRT17^+^), basal (KRT15^+^), proliferating (MKI67^+^), spinous (KRT1^+^/SPRR2E^-^), and supraspinous (KRT1^+^/SPRR2E^+^) (**Figure 3A**; **Supplementary Figure S3B**).

Differential expression analysis comparing PP and NS revealed that supraspinous, spinous, proliferating, and basal keratinocytes exhibited a more pronounced response to psoriasis, evidenced by a larger number of DEGs, whereas follicular keratinocytes displayed a relatively muted response (**Figure 3B**).Further examination of the differential expression of key HKGs and their co-expressed partners indicated that significant changes were primarily observed in the spinous, supraspinous, proliferating, and basal subpopulations, with no notable alterations in follicular keratinocytes (**Figures 3C, D**). This suggests that the co-expression of key HKGs and psoriasis-associated pathogenic genes occurs predominantly in the non-follicular keratinocyte subpopulations.

To explore cell communication differences among keratinocyte subpopulations in psoriasis, we focused on the spinous, supraspinous, proliferating, and basal groups. These subpopulations showed marked gene expression differences between PP and NS, suggesting their critical roles in psoriasis pathogenesis. Ligand-receptor analysis further revealed a significant increase in interactions between these keratinocyte subpopulations and other cell types in psoriasis patients (**Figure 3C**). When treating these four subpopulations as receptor sources, subsequent pathway analysis indicated that the communication networks in psoriasis encompassed several key immune and signaling pathways, including IL - 17, CXCL, midkine (MK), IL - 6, NOTCH, EGF, and MHC-II (**Figure 3D**). Midkine is a heparin-binding growth factor broadly expressed in inflammatory diseases; recent studies have shown that MK regulates VEGF-A expression via the Notch2/HES1/JAK2-STAT5A signaling pathway, thereby exerting critical effects on angiogenesis in psoriasis (34, 35). Collectively, these pathways are intimately linked to chronic inflammation and keratinocyte hyperproliferation in psoriasis.

**Figure 4:**
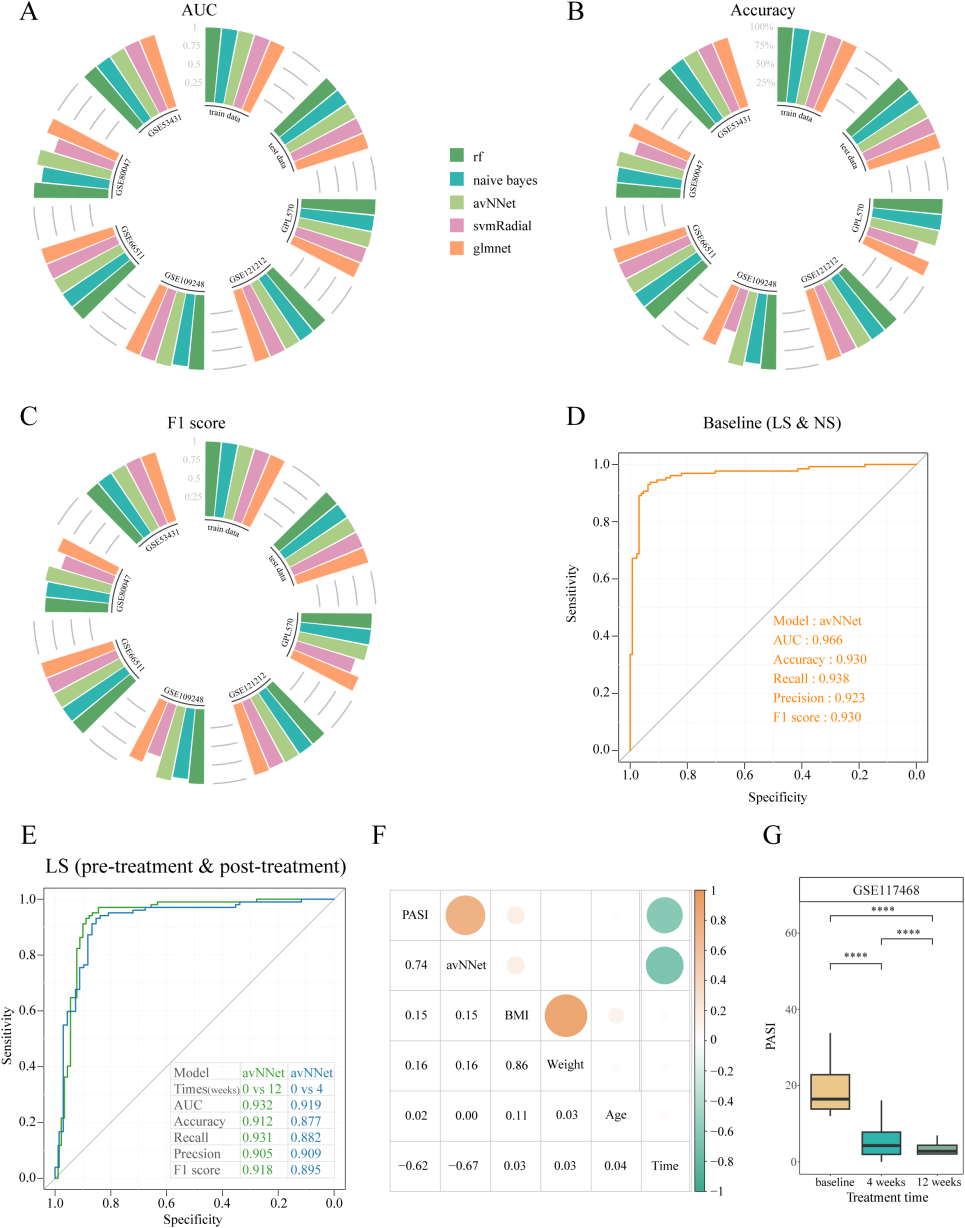
Machine learning models to diagnose psoriasis. **(A)** Circular barplot depicting the AUC values of five machine learning models on different datasets. **(B)** Circular barplot depicting the accuracy of five machine learning models across different datasets. **(C)** Circular barplot depicting the F1 score of five machine learning models across different datasets. **(D)** ROC curves were used to assess the accuracy of the avNNet model in classifying lesional and non-lesional skin. **(E)** ROC curves were used to assess the accuracy of the avNNet model in classifying pre-treatment and post-treatment. **(F)** Heatmap showing correlation between avNNet model scores and clinical indicators. G Box plot comparing PASI across treatment times for the GSE117468 cohort. ****P<0.0001. PASI, psoriasis area and severity index; LS, lesional skin; NS non-lesional skin.

To assess the regulatory effects of the altered cell communication pathways on downstream targets, we employed NicheNet, which integrates expression data with known signaling and gene regulatory networks to predict ligand-target relationships (23). All cells were considered “sending cells,” while the four keratinocyte subpopulations were defined as “receiving cells.” Using NicheNet, we inferred the regulatory potential of ligands on differentially expressed and HKGs within these subpopulations. Our analysis identified four HKGs—RAN, STAT3, EIF5A, and PPARD—whose differential expression was regulated by specific ligands (**Figures 3E, F**, **4A**). Notably, MDK exhibited the highest regulatory potential for RAN, a small GTPase involved in nucleocytoplasmic transport and cell cycle regulation, suggesting that its upregulation may accelerate cell proliferation (36). For STAT3, IL - 6 was the key ligand, consistent with the known role of STAT3 in mediating inflammatory responses via cytokines such as IL - 6, IL - 17A, and EGF (37). IL - 17A emerged as the principal regulator for both EIF5A and PPARD. EIF5A plays a critical role in translation elongation, mRNA stability, and cell growth, and its upregulation via NF-κB pathways may enhance inflammatory mediator production and cellular stress responses (38, 39). PPARD, a nuclear receptor that regulates lipid metabolism, cell proliferation, and differentiation, is crucial for maintaining the epidermal barrier and modulating skin inflammation; its upregulation may represent a negative feedback mechanism to counterbalance IL - 17A-induced inflammation (40). Notably, IL - 17A was the only ligand with regulatory potential for all four HKGs. In addition, IL - 17A regulated key downstream targets including the transcription factor STAT1, chemokines CXCL2, CXCL8, and CCL20, and the structural protein KRT17. These targets play critical roles in controlling cell proliferation, differentiation, and chemotaxis.

Our NicheNet-derived regulatory network delineates the functional interactions between these ligands and HKGs (**Supplementary Figure S4B**). Cellular expression profiling revealed predominant localization of MDK receptors (SDC1/SDC4) in keratinocytes. Notably, IL17A expression exhibited psoriasis-specific restriction to T-cell populations (**Supplementary Figures S4C, D**). Furthermore, psoriatic keratinocytes showed significant upregulation of key transcriptional regulators including CREM, STAT1, and BATF (**Supplementary Figure S4E**).

In summary, in psoriasis, ligand-receptor signaling pathways, especially those involving IL - 17A, significantly modulate the expression of key HKGs in keratinocyte subpopulations. This modulation contributes to abnormal inflammatory responses, immune activation, and keratinocyte hyperproliferation.

### 3.5 Application of the avNNet model based on key housekeeping genes in psoriasis diagnosis and treatment monitoring

Using the GSE54456 dataset, five machine learning models were constructed using 34 key HKGs as features. To further assess model generalizability and potential overfitting, we validated these models on six external datasets, including three merged GPL570 platform datasets. All five models performed exceptionally well on the training and internal test sets, achieving AUC and F1 scores of 1 and a classification accuracy of 100%.

External validation with multiple independent datasets confirmed the stability of our models. Except for the SVM Radial model, which showed prediction accuracies below 75% on some datasets, the other four models achieved accuracies above 80% and F1 scores greater than 0.8 across all datasets. Notably, the avNNet model consistently exceeded 90% prediction accuracy across all datasets (**Figures 4A–C**). These results indicate that the machine learning models based on the identified key HKGs can reliably distinguish between normal and diseased samples, underscoring their potential for molecular-level psoriasis diagnosis.

Building upon avNNet’s exceptional performance, we further validated its clinical utility across two independent cohorts. For skin tissue samples (GSE117468), the model achieved 93% accuracy in discriminating lesional from non-lesional skin (**Figure 4D**), while also demonstrating remarkable sensitivity to treatment response - successfully identifying molecular recovery as early as week 4 post-treatment and accurately classifying clinical responders by week 12 (**Figure 4E**). Notably, the model maintained strong predictive performance (83% accuracy) when applied to PBMC transcriptomes (GSE192867; **Supplementary Figure S5A**), underscoring its robustness across different biological samples. These findings collectively establish avNNet as a sensitive tool for both psoriasis diagnosis and longitudinal monitoring of therapeutic efficacy.

The avNNet-derived HKG signatures enabled stratification of psoriasis patients into distinct high- and low-score phenotypes, revealing significant differential gene expression patterns. GO enrichment analysis revealed that upregulated genes in the high-score group were enriched in antiviral and type I interferon signaling pathways, keratinocyte differentiation, epidermal cell proliferation, and chemokine activity, reflecting the inflammatory and hyperproliferative state characteristic of active psoriasis (41)(**Supplementary Figure S5B**). In contrast, downregulated genes were associated with extracellular matrix organization, collagen metabolism, epidermis development and wound healing, suggesting reduced tissue remodeling and normalization of keratinocyte hyperproliferation in the low-score group (42) (**Supplementary Figure S5C**).

Finally, we observed a significant correlation between the avNNet model score and the clinical Psoriasis Area and Severity Index (PASI) (correlation = 0.74, P = 4.4e-47) (**Figures 4F, G**). This strong correlation indicates that the molecular-level scoring provided by the avNNet model closely reflects traditional clinical assessment metrics, offering a novel approach for evaluating psoriasis progression and treatment outcomes.

## 4 Discussion

Our study systematically elucidates the critical role of HKGs in psoriasis pathogenesis through integrative transcriptomic analysis and machine learning modeling. By combining bulk and single-cell transcriptomic analyses, we identified 34 HKGs that exhibit dysregulated expression patterns tightly linked to keratinocyte dysfunction and immune dysregulation. These findings not only expand our understanding of psoriasis mechanisms beyond canonical pathways but also establish a novel framework for molecular diagnosis and therapeutic monitoring.

In this study, we draw on the “moonlighting protein” paradigm to redefine a subset of HKGs as “dual‐function regulators,” recognizing that a single gene product can fulfill disparate roles depending on cellular context (43, 44). Specifically, under homeostatic conditions, these regulators sustain essential cellular processes, while in pathological states such as psoriasis, they perform additional regulatory functions. This redefinition leverages the comprehensive reanalysis by Eisenberg and Levanon, who cataloged 3,804 high‐confidence HKGs based on their ubiquitous and stable expression across human tissues (6). Our results reveal that, within psoriatic lesions, a subset of these genes not only exhibit disrupted expression stability but also actively participate in inflammatory signaling and keratinocyte differentiation pathways, thus extending well beyond their canonical housekeeping roles. Previous work has documented context‐dependent repurposing of classical HKGs. For example, GAPDH, beyond its glycolytic function, also modulates transcription and apoptosis (45). Here, we demonstrate that in psoriasis, HKGs such as STAT3, PPIF, and EIF5A are co‐opted by the IL‐17/IL‐6 signaling axis to drive keratinocyte activation. These findings underscore a conserved mechanism of functional repurposing, whereby traditionally “housekeeping” molecules become integral components of disease‐specific regulatory circuits.

In multiple cohorts, our study identified 34 highly conserved dysregulated HKGs, underscoring their critical role in psoriasis. Notably, STAT3, EIF5A, and RAN emerge as pivotal nodes in keratinocyte activation driven by IL - 17A signaling. Overexpression of RAN has been associated with increased cellular proliferation in breast cancer (46), while persistent STAT3 activation has long been implicated in psoriasis (47). Moreover, RAN appears to act synergistically with STAT3 by modulating its nuclear import (48). As a key translation factor, EIF5A, and in particular its isoform EIF5A2, is overexpressed in various cancers (49). These mechanisms are consistent with the hyperproliferative and inflammatory phenotype observed in psoriatic keratinocytes. We also observed downregulation of CD81, which can enhance NF-κB–mediated inflammation and upregulation of PARP9, which is a known promoter of proinflammatory gene expression (50, 51). These findings support prior studies linking HKG dysregulation to inflammatory processes. Additionally, our single-cell data reveal that HKG dysregulation is predominantly confined to non-follicular keratinocyte subpopulations (basal, spinous, supraspinous, and proliferative cells), which are the primary drivers of epidermal hyperplasia. This spatial specificity emphasizes the compartmentalized nature of HKG-driven pathology in psoriasis.

The co-expression shifts of HKGs with psoriasis-associated genes (e.g., DEFB103A/B, S100A7A, IL36RN) further demonstrate their regulatory influence. The transition from negative to positive correlations in disease states suggests that HKGs may act as molecular rheostats, rewiring transcriptional networks to sustain inflammation. For instance, the interaction between DCUN1D3 and IL36RN, a key antagonist of IL - 36 receptor signaling, implies a role in modulating IL - 36 dependent epidermal barrier dysfunction, a mechanism recently implicated in pustular psoriasis (52). These findings bridge the gap between HKGs and established pathogenic pathways, positioning them as amplifiers of disease-specific signals.

The avNNet model’s exceptional performance (AUC >0.9 across datasets) underscores the diagnostic utility of HKGs. Unlike previous biomarker panels that rely on immune-specific genes, our HKG-based approach captures keratinocyte-centric molecular shifts, enabling earlier detection of subclinical inflammation. The model distinguishes treated from untreated skin with 93% accuracy and shows a strong correlation with PASI (ρ = 0.74), positioning it as a robust tool for monitoring therapeutic response. Notably, the model detected molecular recovery at 4 weeks post-treatment, a timepoint when clinical improvement is often ambiguous, suggesting its potential to guide personalized treatment escalation. The superior performance of the avNNet model can be attributed to its ensemble averaging approach, which aggregates the outputs of multiple individual neural networks. This strategy not only markedly enhances overall accuracy and prediction stability but also reduces reliance on hyperparameter tuning for any single network, thereby simplifying model optimization (53). Furthermore, by integrating multiple weak learners, avNNet mitigates the risk of overfitting. In contrast, the inferior performance of the SVM Radial model likely stems from its dependence on two critical hyperparameters, the penalty parameter C and kernel width γ, where inappropriate combinations can easily lead to underfitting or overfitting (54). This underscores the necessity of selecting algorithms specifically tailored to capture HKG expression dynamics, as these biological patterns often involve complex nonlinear interactions that are best captured by neural network architectures.

While our findings are compelling, several limitations warrant consideration. First, the reliance on pre-treatment lesional skin samples may overlook dynamic HKG changes during disease flares or treatment. Second, SCO2 and NOP2’s inconsistent expression in single-cell data raises questions about technical variability versus biological context-dependency. Third, our ligand-receptor analyses were based solely on transcriptomic inference without protein-level or functional validation, such as phospho-protein assays or cytokine blockade experiments. Additionally, while our avNNet model demonstrated high diagnostic accuracy and correlation with PASI, its ability to distinguish psoriasis subtypes and capture immune-driven heterogeneity remains to be validated. These analytical constraints, common in large-scale transcriptomic studies, highlight important directions for future investigation. As multi-omics datasets become more accessible, subsequent studies could further validate these networks, while expanded cohorts would enable refined subtype analyses. Looking ahead, the implementation of spatial transcriptomics could precisely map HKG activity across epidermal-dermal microenvironments, while CRISPR-based functional screens would help establish causal relationships. Such advances would significantly accelerate the translation of our findings into clinical applications, particularly for personalized treatment strategies in psoriasis management.

## 5 Conclusion

By redefining HKGs as active contributors to psoriasis pathogenesis, this study challenges the traditional view of HKGs as mere experimental controls. Our multi-level transcriptomic analysis reveals their dual role as both stabilizers of cellular homeostasis and drivers of disease-specific networks, offering new therapeutic targets. The avNNet model further bridges molecular insights to clinical practice, providing a scalable tool for precision dermatology. These advances pave the way for HKG-directed therapies and biomarker-driven management of psoriasis.

## Data Availability

Publicly available datasets were analyzed in this study. All data supporting the findings of this study are publicly available in the GEO database under the following accession numbers: GSE54456, GSE14905, GSE226244, GSE182740, GSE121212, GSE109248, GSE66511, GSE80047, GSE53431, GSE117468, GSE173706 and GSE192867. These datasets can be accessed directly through the GEO repository at https://www.ncbi.nlm.nih.gov/geo/.
