# Supplementary material for: Housekeeping gene dysregulation in psoriasis: integrative multi‐cohort and single‐cell analysis reveals keratinocyte‐centric molecular mechanisms and diagnostic biomarkers
Source: Front Immunol. 2025 Sep 1;16:1601705. doi: 10.3389/fimmu.2025.1601705 (PMC12433973; doi:10.3389/fimmu.2025.1601705)
Supplement: Supplementary file 2 [file Table1.docx]

Supplementary Material

1. **Supplementary Figures and Tables**

## Supplementary Figures


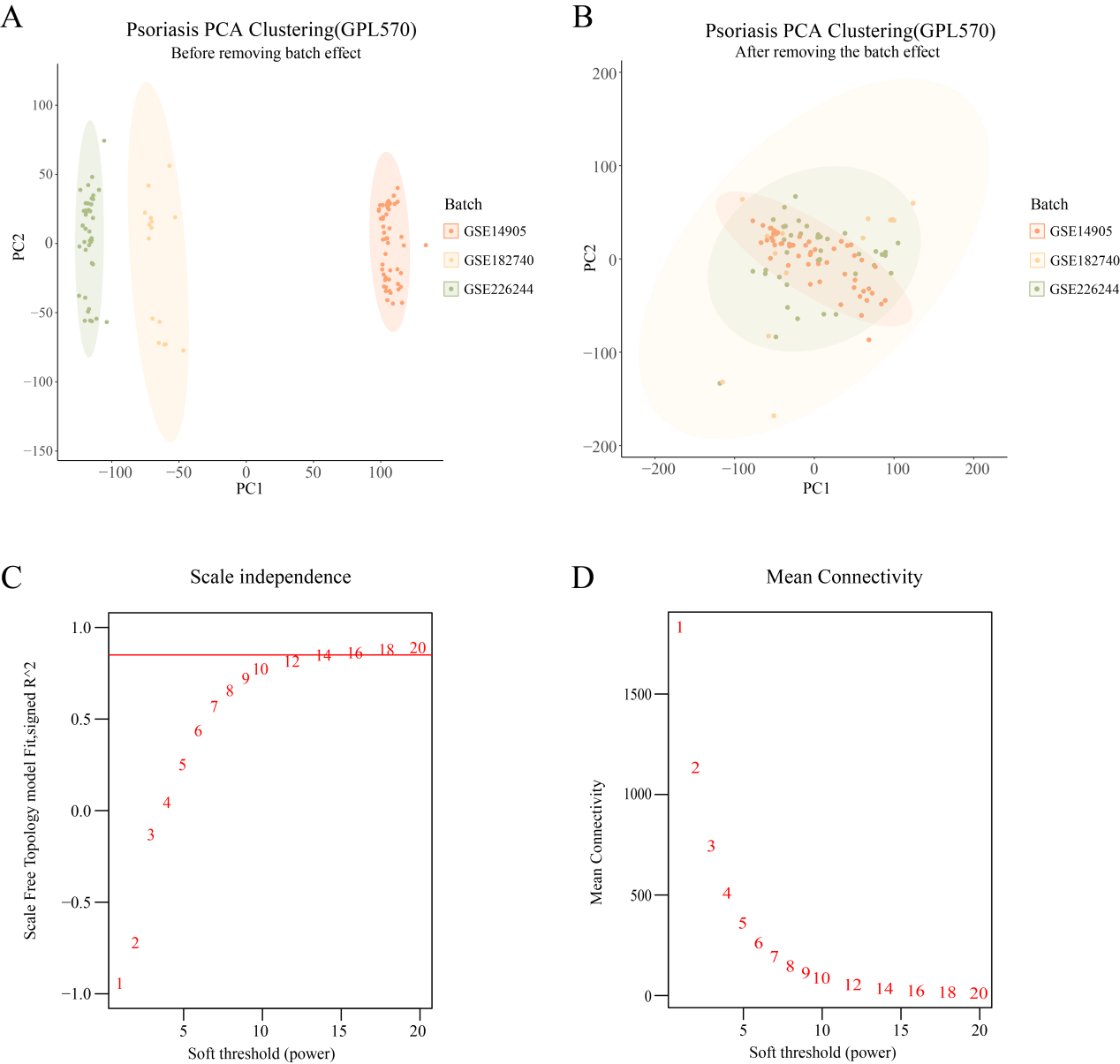


**Figure S1** Batch effect removal and the selection of soft thresholds. **(A)** PCA plot showing the GPL570 cohort before removal of batch effects. **(B)** PCA plot showing the GPL570 cohort after removal of batch effects. **(C)** Scale independence in the [GPL9](https://www.ncbi.nlm.nih.gov/geo/query/acc.cgi?acc=GPL570)052 cohort. **(D)** Mean connectivity in the [GPL9](https://www.ncbi.nlm.nih.gov/geo/query/acc.cgi?acc=GPL570)052 cohort.


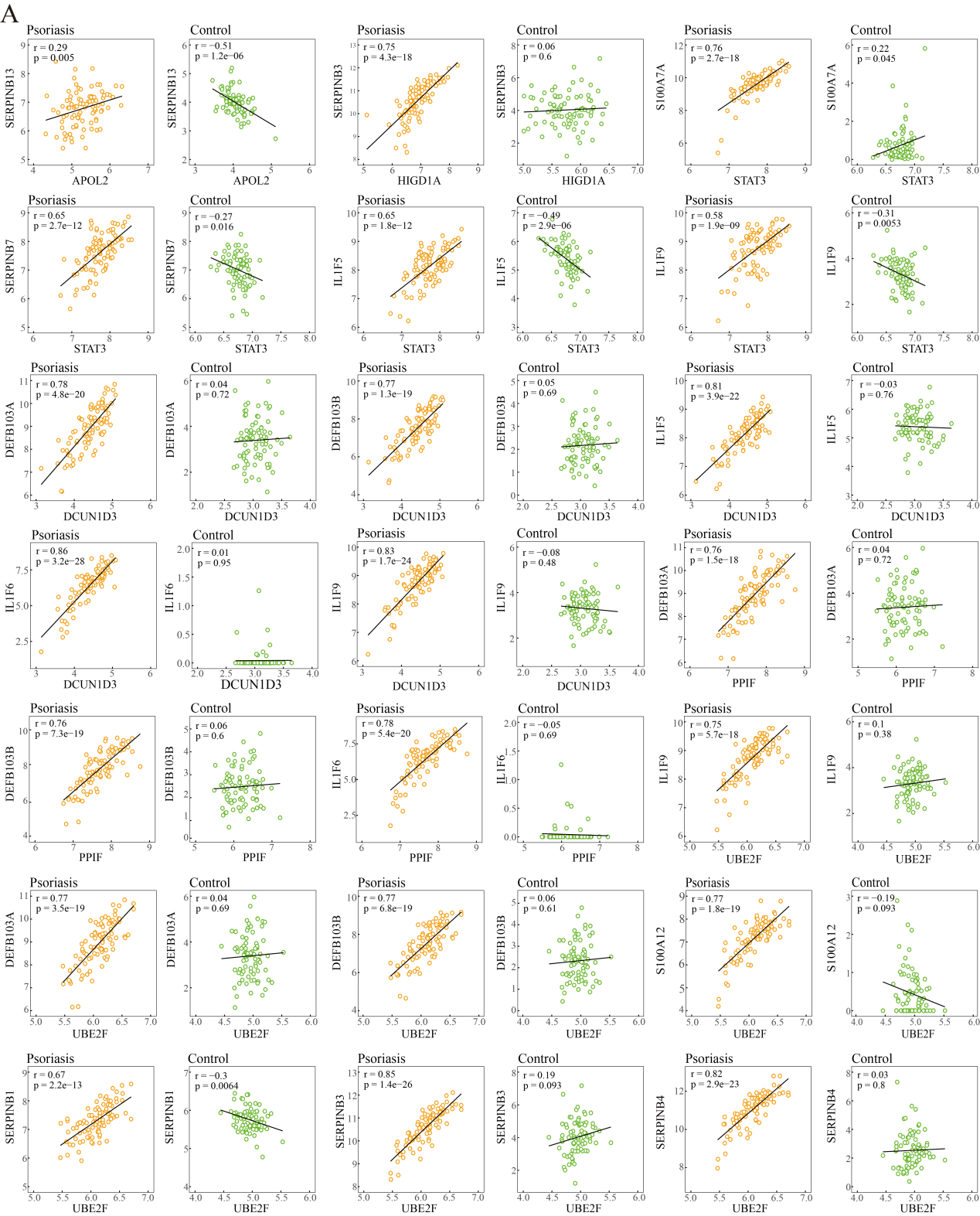


**Figure S2** Correlation coefficients between housekeeping genes and psoriasis genes. **(A)** Scatterplots depicting changes in the correlation between housekeeping genes and pathogenic genes in psoriasis patients and controls.


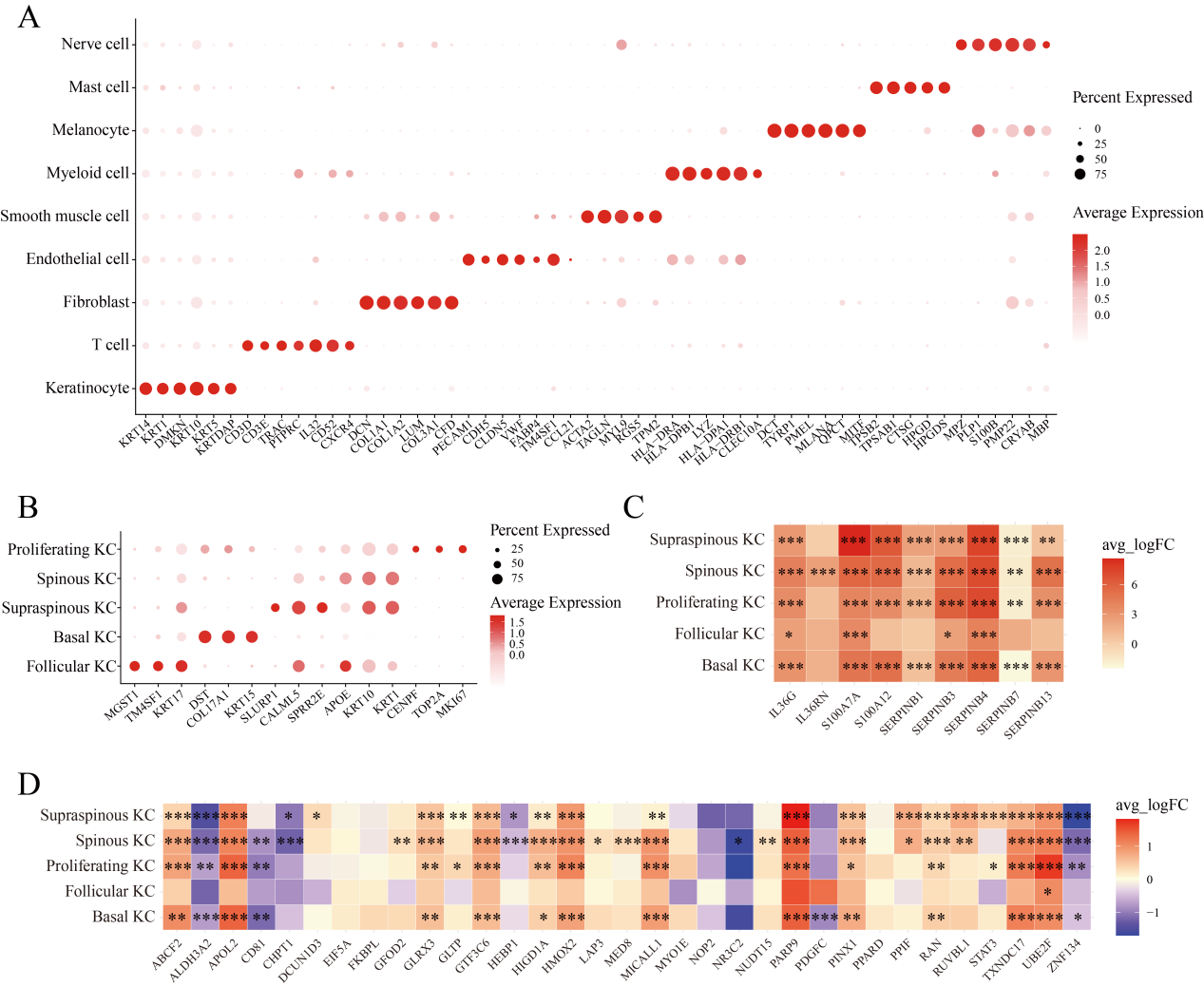


**Figure S3** Gene expression profiles in different cell types. **(A)** Dot plot showing expression of marker genes for each cell type. **(B)** Dot plot showing expression of marker genes for each cell type. **(C)** Heatmap showing differential expression of genes in different cell types. **(D)** Heatmap showing differential expression of genes in different cell types.


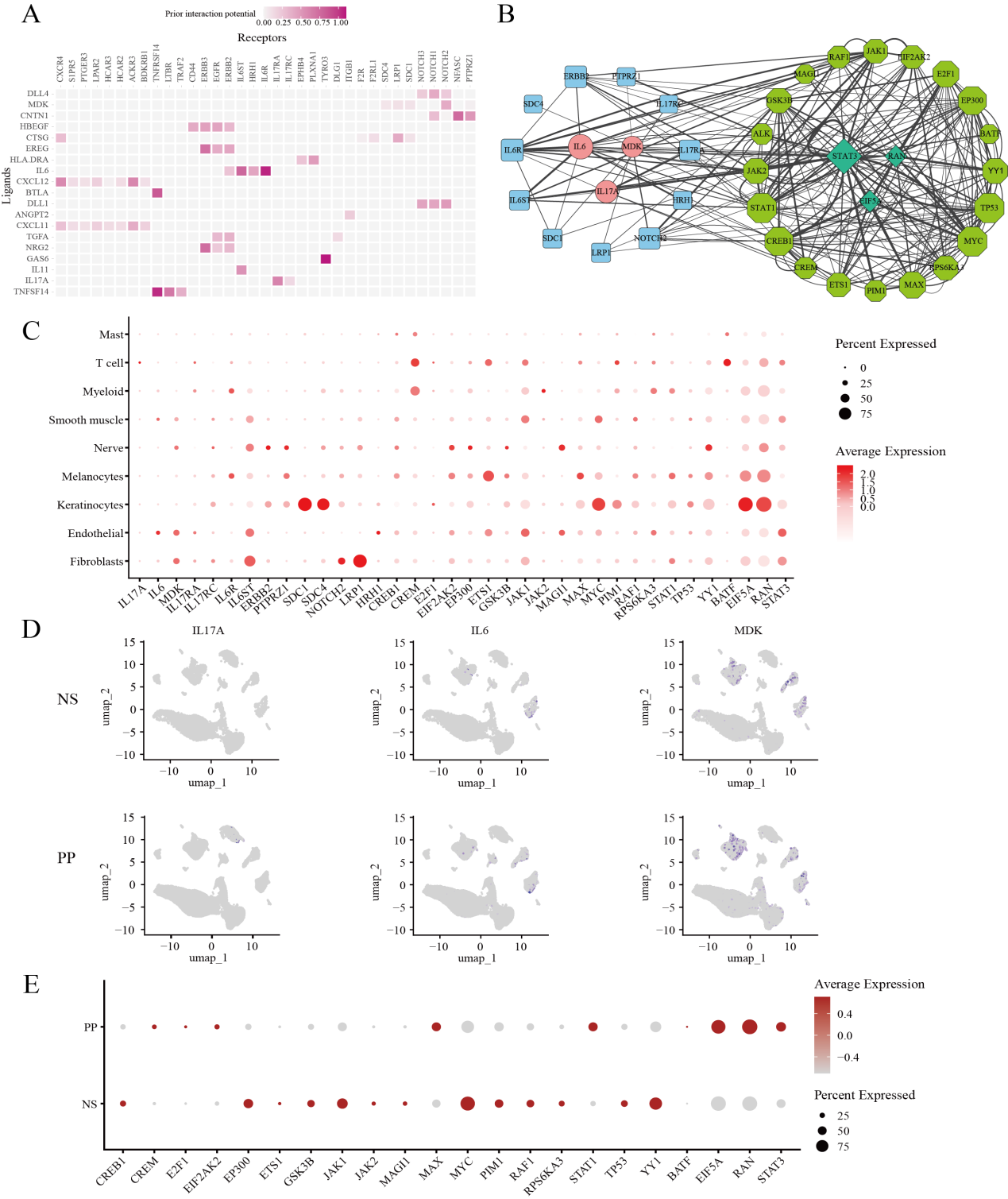


**Figure S4** Ligand-to-target gene regulatory network diagram and expression of genes within this network **(A)** NicheNet ligand-receptor analysis: matrix showing predicted ligand-receptor interactions and their interaction strength across four keratinocyte subpopulations. **(B)** Network diagram showing the predicted regulatory relationships of the three ligands with housekeeping genes. **(C)** Dot plot showing expression of genes for each cell type. **(D)** UMAP plot showing the expression of IL17A, IL6, and MDK in normal and psoriatic skin samples. **(E)** Dot plot showing the expression of downstream genes in keratinocytes from psoriatic and normal skin samples.


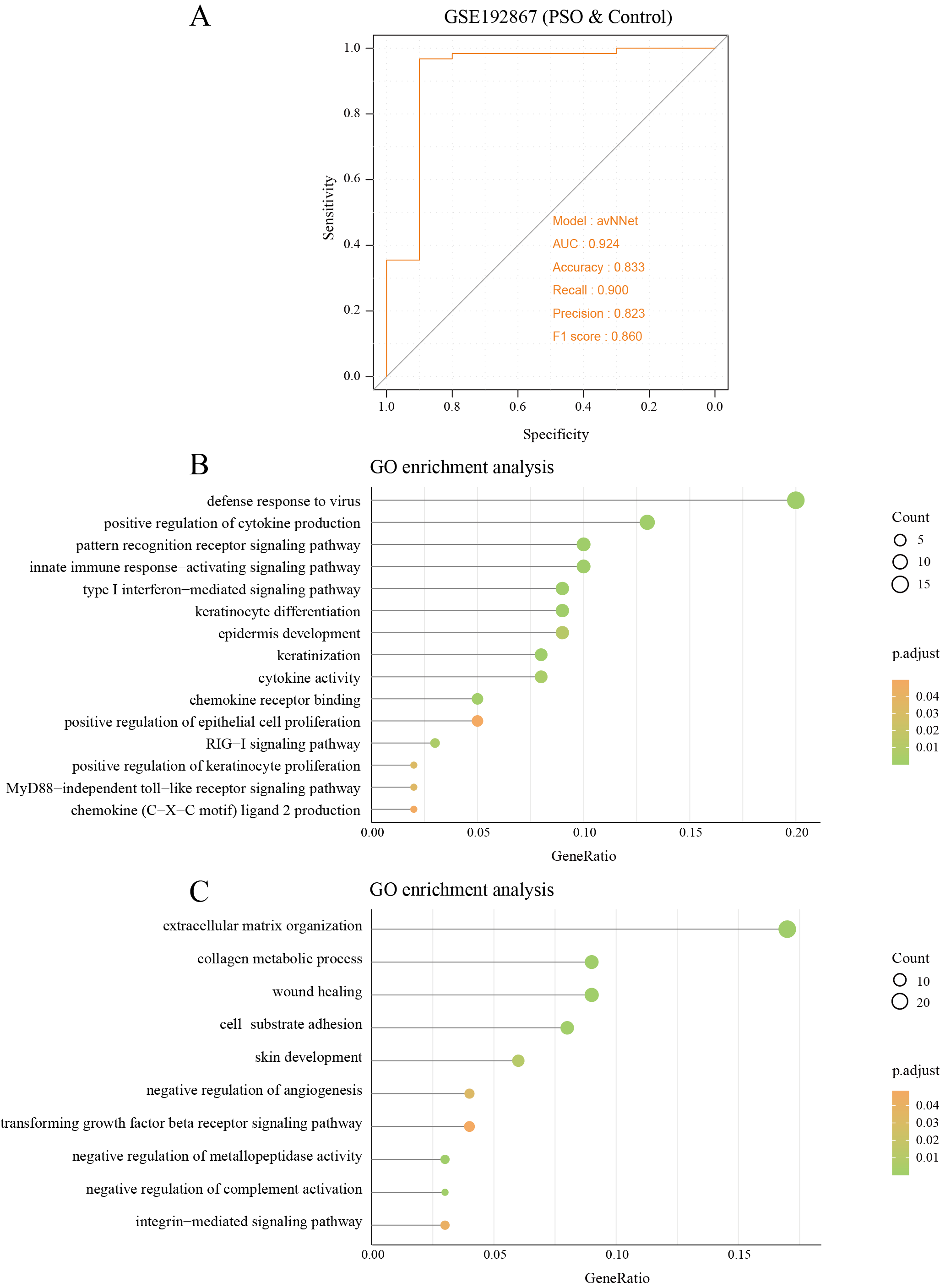


**Figure S5** avNNet Classification of PBMC Samples and GO Enrichment Analysis of Score-Stratified Genes from Skin Samples. **(A)** ROC curves were used to assess the accuracy of the avNNet model in classifying psoriasis and control. **(B)** GO enrichment analysis of the up-regulated genes in the high-score group. **(C)** GO enrichment analysis of the down-regulated genes in the high-score group.
